# Tapering of biological treatment in autoinflammatory diseases: a scoping review

**DOI:** 10.1186/s12969-022-00725-3

**Published:** 2022-08-13

**Authors:** Tatjana Welzel, Lea Oefelein, Marinka Twilt, Marc Pfister, Jasmin B. Kuemmerle-Deschner, Susanne M. Benseler

**Affiliations:** 1 Pediatric Rheumatology and autoinflammation reference center Tuebingen (arcT), Department of Pediatrics, Member of the European Reference Network for rare or low prevalence complex diseases, network Immunodeficiency, Autoinflammatory and Autoimmune Diseases (ERN RITA), University Children`s Hospital Tuebingen, Tuebingen, Germany; 2 Pediatric Pharmacology and Pharmacometrics, Pediatric Rheumatology, University Children`s Hospital Basel (UKBB), University of Basel, Spitalstrasse 33, CH 4031 Basel, Switzerland; 3 Rheumatology, Department of Pediatrics, Alberta Children`s Hospital, Cumming School of Medicine, Alberta Children’s Hospital Research Institute, University of Calgary, Calgary, AB Canada

**Keywords:** bDMARDs, AID, Treat to target, Pharmacokinetics, Pharmacodynamics, Personalized treatment, Dose-concentration-response relationship

## Abstract

**Background:**

Biological treatment and treat-to-target approaches guide the achievement of inactive disease and clinical remission in Autoinflammatory Diseases (AID). However, there is limited evidence addressing optimal tapering strategies and/or discontinuation of biological treatment in AID. This study evaluates available evidence of tapering biological treatment and explores key factors for successful tapering.

**Methods:**

A systematic literature search was conducted in Embase, MEDLINE, Cochrane Database of Systematic Reviews and Cochrane Central Register of Controlled Trials using the OVID platform (1990-08/2020). Bibliographic search of relevant reviews was also performed. Studies/case series (n ≥ 5) in AID patients aged ≤ 18 years with biological treatment providing information on tapering/treatment discontinuation were included. After quality assessment aggregated data were extracted and synthesized. Tapering strategies were explored.

**Results:**

A total of 6035 records were identified. Four papers were deemed high quality, all focused on systemic juvenile idiopathic arthritis (sJIA) (1 open-label randomized trial, 2 prospective, 1 retrospective observational study). Biological treatment included anakinra (*n* = 2), canakinumab (*n* = 1) and tocilizumab (*n* = 1). Strategies in anakinra tapering included alternate-day regimen. Canakinumab tapering was performed randomized for dose reduction or interval prolongation, whereas tocilizumab was tapered by interval prolongation. Key factors identified included early start of biological treatment and sustained inactive disease.

**Conclusion:**

Tapering of biological treatment after sustained inactive disease should be considered. Guidance for optimal strategies is limited. Future studies may leverage therapeutic drug monitoring in combination with pharmacometric modelling to further enhance personalized “taper-to-target” strategies respecting individual patients and diseases aspects.

**Supplementary Information:**

The online version contains supplementary material available at 10.1186/s12969-022-00725-3.

## Background

Autoinflammatory Diseases (AID) encompass a heterogeneous group of rare lifelong diseases often already manifesting in infancy and early childhood. AID are typically caused by pathogenic gene variants resulting in excessive production of pro-inflammatory cytokines [1]. The hallmark of AID are clinical features of systemic and organ inflammation and laboratory evidence of innate immune system activation ultimately leading to significant morbidity with organ damage and mortality [2, 3]. Thus, early diagnosis and rapid start of effective therapies are crucial. Biological treatments with anti-cytokine monoclonal antibodies (mAb) and receptor antagonists targeting the activated pathways, including Interleukin (IL)-1 and IL-6 inhibitors are highly effective in AID [4–6]. Optimal control of disease activity in AID can be achieved through treat-to-target (T2T) approaches [7–9]. Long-term treatment results in sustained remission on medication and prevention of organ damage.

However, this comes at a price: children and youth particularly dislike daily, weekly or monthly injections/infusions. Dreaded complications mainly include increased risk of infections, injection-site reactions, and gastrointestinal adverse events [4–6]. Furthermore, the costs of biological treatments constitute a significant financial burden [10].

Both individual and societal burden of AID raise the question of the optimal duration of biological treatment. Do patients require lifelong treatment? Is there a robust, “true” clinical and biological remission in AID? Can biological treatment be tapered or even discontinued and how? What are the key factors associated with sustained remission? When is it safe to consider tapering/discontinuing biological treatments and which strategy is optimal?

To date, limited evidence is available, and no recommendations have been published addressing optimal tapering/discontinuation of biological treatment in AID. Therefore, the aims of the study were (1) to evaluate and synthetize the available evidence of biological treatment tapering/discontinuation in children and youth with AID and (2) to explore key factors that may inform successful strategies.

## Methods

### Data sources and searches

A systematic literature search was conducted following the Preferred Reporting Items for Systematic Reviews and Meta-Analyses guidelines [11] and methods guidelines [12]. Comprehensive searches were conducted using the OVID platform in the following databases: Embase (1990-08/2020); MEDLINE Epub ahead of print, In-process and other non-indexed citations (08/2020); MEDLINE without revisions (1996-08/2020); Evidence-Based Medicine (EBM) Reviews: Cochrane Database of Systematic Reviews (2005-08/2020); and EBM Reviews: Cochrane Central Register of Controlled Trials (08/2020). MeSH and search terms included hereditary autoinflammatory diseases, cryopyrin associated periodic syndromes (CAPS), familial Mediterranean fever (FMF), mevalonate kinase deficiency (MKD), hyper-IgM immunodeficiency, systemic juvenile idiopathic arthritis (sJIA), tumor necrosis factor receptor-associated periodic syndrome (TRAPS), adult-onset still’s disease (AOSD), in addition to all terms related to treatment, stopping of treatment and therapeutics (details of search protocol see Additional file 1: Table S1). Furthermore, a bibliographic search of relevant publications was performed to identify additional studies.

### Study selection

Original articles included retrospective, prospective and survey studies, randomized controlled studies and case series, if the sample size was ≥ 5. All references were screened by perusing titles and abstracts for inclusion criteria by two independent reviewers. In case no abstract was available, the reference went automatically to full-text review. Studies were deemed eligible and were selected for full-text review: (1) disease of interest, (2) age ≤ 18 years, (3) biological treatment, (4) information on intervention (tapering/discontinuation), (5) published in English. The review excluded: (1) non-human studies (laboratory or animal studies), (2) non-original research (reviews, editorials, letters to the editor, expert opinions) and (3) abstracts only available in conference supplements. The primary reason for exclusion was documented for each excluded reference.

### Quality assessment

Selected studies underwent a standardized quality assessment (Additional file 1: Table S2). Items included the selected treatment, methods and reasons for tapering/discontinuation. For each item, the maximum score was 1 resulting in a total score of 6. Publications were rated “high quality”, if they obtained a total score of ≥ 4 including ≥ 2 of the four designated “essential questions” (Additional file 1: Table S2). All selected articles were assigned an Oxford Level of Evidence based on study type and quality [13].

### Evidence synthesis, data extraction and analysis

For studies determined high quality full-text were reviewed by two independent reviewers, discrepancies were resolved by consensus. Aggregate data were extracted from each study by two independent reviewers. Variables included study design, AID subtype, gender, criteria for inactive disease/remission, biological treatment (drug, dose, frequency), intervention (tapering/discontinuation), tapering/discontinuation strategy, number of patients with intervention, number of patients with successful tapering/discontinuation, and duration of follow-up. Studies were analysed and compared focusing on the decision to taper/discontinue, the strategy and the effectiveness of tapering/discontinuation.

### Definitions

Wallace et al. defined the state of inactive disease and clinical remission (CR) for JIA including sJIA [14]. Inactive sJIA was defined as: no joints with active arthritis, no fever, rash, serositis, splenomegaly, or generalized lymphadenopathy attributable to JIA, no active uveitis, normal erythrocyte sedimentation rate or C-reactive protein (CRP) and physician’s global assessment (PGA) indicating no disease activity [14]. All criteria must be met for the disease status “inactive disease”. CR on medication was defined as an inactive disease status for ≥ 6 consecutive months while the patient is receiving medication. CR off medications is considered inactive disease for ≥ 12 consecutive months while the patient is not receiving medication [14].

For FMF, CAPS, TRAPS and MKD disease activity is assessed using the validated autoinflammatory disease activity index (AIDAI) [15]. The AIDAI contains 12 items including fever (> 38 °C), overall symptoms and organ-specific AID symptoms, scored as 1 (present/yes) or 0 (absent/no). The maximum daily score is 12 with a cumulative monthly score ranging from 0 to 372 (31-day month). Inactive disease is defined as an AIDAI score below 9/month [15].

In clinical trials, disease activity is commonly assessed by the PGA and patients/parent global assessment (PPGA) recorded on a 10 cm visual analogue scale (VAS) with 0 (no disease activity) and 10 (maximum disease activity). Furthermore, laboratory parameters, such as CRP and/or serum amyloid A (SAA) are measures of disease activity used in trials. Treatment responses are defined by a composite index of PPGA/PGA plus inflammatory parameters [4, 5].

## Results

### Study selection

**Fig. 1:**
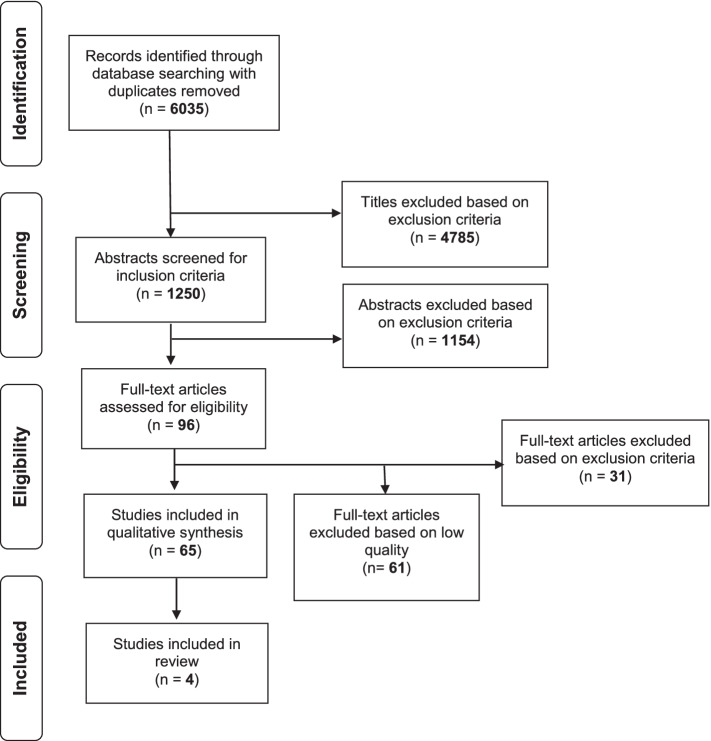
Flowsheet study selection

The search identified 6035 records after duplicate removal. After title selection, 1250 references were selected, and abstracts were reviewed revealing 96 articles eligible for full-text review. Another 31 articles were removed based on exclusion criteria (Fig. 1). After quality assessment, four manuscripts [16–19] were deemed of high quality and included in the final evidence synthesis (Fig. 1). Data of all four studies for the variables of interest were summarized in Table 1.

**Table 1:** Overview of tapering studies

Author	Study design	Disease	Gender (%, female)	Biological treatment, add-on medication	Criteria for inactive disease/remission	Criteria for start of tapering/discont.	Reported strategy of treatment tapering/discont.	Patients started tapering/discont. (n)	Patients with successful tapering (n)	Patients with successful discont. (n)	Follow- up time
**Vastert et al.** [19]	Prospective cohort study	sJIA	35%	Anakinra 2 mg/kg daily, max. 100 mg daily, no cDMARDs	Wallace criteria or Adapted ACR Pedi 90 response	Inactive disease or adapted ACR Pedi 90 responses at 3 months	Alternate-day regimen for 1 month, followed by discont., 2nd tapering attempt 3–6 months later	15	7 (first attempt), plus additional 4 (second attempt)	11	at least 12 months, mean follow-up 2.67 years (1-4.5 years)
**Ter Haar et al. **[18]	Prospective cohort study	sJIA	40%	Anakinra 2 mg/kg daily (max. 100 mg daily) (incomplete response: 4 mg/kg daily; max 200 mg daily), no cDMARDS	Wallace criteria	Inactive disease at 3 months	Alternate-day regimen for 1 month, followed by discont.,	33	31	18	Up to 5 years
**Quartier et al. **[17]	IIIb/IV Open-label RCT	sJIA	52%	Canakinumab 4 mg/kg q4w, no cDMARDs	Wallace criteria	Inactive disease for 6 months	**Arm 1: dose reduction** three steps approach (each 24 weeks): 4 mg/kg/q4w to 2 mg/kg/q4w to 1 mg/kg/q4w to discontinuation **Arm 2: interval prolongation** three steps approach (each 24 weeks): 4 mg/kg/q4w to 4 mg/kg/q8w to 4 mg/kg/q12w to discontinuation	**Arm 1** 38 **Arm 2** 37	**Arm 1** 27/26/17* **Arm 2** 31/30/8* *step 1/2/3	**Arm 1** 17 **Arm 2** 8	24 weeks
**Kostik et al. **[16]	Retro-spective obser-vational study	sJIA	57%	Tocilizumab 12 mg/kg (< 30 kg) or 8 mg/kg (≥ 30 kg) q2w or q4w, add-on cDMARDs	Wallace criteria	Inactive disease for ≥ 12 months	Interval prolongation up to q5w for 3–4 months, then q6w for 3 months, if no clinical and laboratory signs of disease activity treatment was discontinued	7	7 (of these 3 with MTX)	7 (of these 3 with MTX)	No MTX: 1078 days (IQR 848–1217), MTX: 918 days (IQR 508–1078)

*Abbreviations: cDMARDs *conventional Disease Modifying Antirheumatic Drugs, *discount. *discontinuation, *sJIA *systemic juvenile idiopathic arthritis, *mg* milligram, *kg *kilogram, *ACR Pedi 90 responses *American College of Rheumatology Pediatric Response Criteria 90, *q4w *every 4 weeks; *q8w *every 8 weeks, *q12w* every 12 weeks, *RCT* randomized controlled trial, *MTX* methotrexate, *IQR* interquartile ranges

### Study characteristics

The four identified studies included one open-label randomized trial [17], two prospective [18, 19] and one retrospective observational study [16]. The AID disease of interest across all studies was sJIA (*n* = 4) [16–19]. No studies were identified for other AID. Treatment regimens included IL-1 inhibition with anakinra (*n* = 2) and canakinumab (*n* = 1) [17–19] and IL-6 inhibition with tocilizumab (*n* = 1) [16].

### Treatment regimens

- IL-1 inhibition: Anakinra was commonly given at dosing regimens of 2 (max. 100 mg/day)-4 mg/kg/day (max. 200 mg/day) subcutaneous (s.c.) [18, 19]. Therapy with canakinumab was typically dosed at 4 mg/kg s.c. every four weeks (q4w) [17].
- IL-6 inhibition: Dosing of tocilizumab was 12 mg/kg (< 30 kg) and 8 mg/kg (≥ 30 kg) administered intravenous (IV) every two weeks (q2w) in case of severe disease activity and q4w in case of milder disease activity [16].

### Treatment taper or discontinuation in sJIA

#### Anakinra

Vastert et al. [19] analysed tapering and discontinuation of anakinra in children with newly diagnosed sJIA between 2008 and 2012. In this prospective single-center cohort study, all patients were treated with anakinra 2 mg/kg daily (maximum 100 mg) s.c. for three months. An attempt was made to switch to anakinra alternate-day regimen at the same dose for one month, if children demonstrated an adapted ACR Pedi 90 response [20] or clinically inactive disease at the three-months’ time point. Subsequently, anakinra was discontinued one month later, if at least an adapted ACR Pedi 90 response was maintained. Children, who experienced disease symptoms on alternate-day anakinra were switched back to daily anakinra. Tapering was re-attempted 3 to 6 months later. In 7/15 patients (47%) tapering was successful and ultimately anakinra could be discontinued. Eight children (53%) experienced relapses during tapering. In those children, the daily anakinra regimen was restarted. Ultimately, 11/15 sJIA patients (73%) were able to stop anakinra while maintaining inactive disease.

The Utrecht group further expanded their cohort of new onset sJIA patients treated early with anakinra to 2017 [18]. In addition, children diagnosed with arthralgia but with no overt arthritis at diagnosis and those with suspected sJIA after exclusion of other differential diagnoses were included. Anakinra was tapered, if patients had evidence of inactive disease according to the modified Wallace criteria [14] after three months of treatment. This approach was consistent with the one chosen in the initial cohort [19]. Anakinra was restarted if a disease flare occurred. Children that failed multiple attempts of tapering anakinra were offered canakinumab treatment. A total of 42 children were treated with anakinra. Anakinra tapering was commenced in 33 children after a median period of 3.7 months. Of these, 2/33 children (6%) experienced recurrent disease activity while anakinra was being tapered. All continued daily IL-1 blockade. Anakinra discontinuation was possible in 31/33 sJIA patients (94%). A total of 29 did so within the first year of therapy. After anakinra discontinuation, 18/31 patients (58%) remained in remission without treatment for years. A total of 13/31 patients (42%) experienced flares with a median time to flare of five weeks (IQR 3 weeks, 5 months). All 13 children re-started anakinra. In 3/13 patients anakinra discontinuation was successful within the first year of therapy. Ultimately, 18/33 (54%) children maintained inactive disease off medication long-term.

#### Canakinumab

Quartier et al. [17] performed a two-part phase IIIb/IV open-label randomized trial of 182 sJIA patients, including 98/182 (54%) canakinumab-naïve patients. At enrollment, all children were treated with canakinumab 4 mg/kg/q4w s.c. Canakinumab responders were then randomized 1:1 to two different tapering regimes. In arm 1, canakinumab was tapered by dose reduction, in arm 2 by interval prolongation. Each tapering regime was subdivided into three steps with a duration of 24 weeks for each step. Patients sustaining inactive disease in one step were able to enter the subsequent one.

A total of 75/182 patients (41%) achieved CR for a minimum of 6 months on Canakinumab according to Wallace criteria [14]. In arm 1 (N = 38 patients), canakinumab doses were reduced from 4 mg/kg/q4w s.c. to 2 mg/kg/q4w s.c. and then to 1 mg/kg/q4w s.c., followed by discontinuation. In arm 2 (N = 37 patients), 4 mg/kg s.c. canakinumab dosing intervals were extended from four to eight weekly (q8w) for 24 weeks, then to every twelve weeks (q12w), followed by discontinuation. In dose reduction arm 1, CR was maintained in 27/38 patients (71%) when canakinumab was decreased to 2 mg/kg/q4w, while 29% experienced a disease flare. When the dose was reduced further to 1 mg/kg/q4w, 26/38 patients (68%) remained inactive. Ultimately, 17/38 patients (45%) in arm 1 were able to discontinue canakinumab. In arm 2 (interval prolongation), CR was maintained in 31/37 patients (84%) at canakinumab 4 mg/kg q8w, 16% flared. At the 12-weekly interval, 30/37 patients (81%) remained inactive. Ultimately, 8/37 sJIA patients (22%) were able to discontinue canakinumab. In arms 1 and 2 combined a total of 25/75 sJIA patients (33%) were able to discontinue canakinumab while maintaining inactive disease, 17/38 (45%) in arm 1 (dose reduction) and 8/37 patients (22%) in arm 2 (interval prolongation) [17].

#### Tocilizumab

Kostik et al. [16] retrospectively studied 37 children with sJIA treated with tocilizumab. The median time from diagnosis to start of tocilizumab was 36 months (range 10.7-97.0). The administration interval was either q2w or q4w depending on disease severity assessed by the treating physician. A total of 12/37 (32%) achieved inactive disease according to Wallace criteria [14]. Tocilizumab was tapered and discontinued in seven patients; all had a least a 12-month tocilizumab course and were previously weaned off corticosteroids and cyclosporine A. Add-on treatment with methotrexate (MTX) was allowed. Tocilizumab was tapered by infusion interval prolongation, reported as infusion administration every five weeks (q5w) for three to four months, and then every six weeks (q6w) for three months. Tocilizumab was discontinued if the patient had neither clinical nor laboratory signs of sJIA. After stopping tocilizumab, the authors reported that 4/7 patients (57%) remained inactive without MTX for a median of 1078 days (848–1217 days). The remaining three patients received add-on MTX and stayed in remission for a median of 918 days (508–1078). Ultimately, tocilizumab was successfully discontinued in all seven patients.

### Non-sJIA

No studies on tapering/discontinuation of biological treatment in children with CAPS, TRAPS, FMF, MKD/HIDS, AOSD or other AID are available to date.

## Discussion

This scoping review is the first to synthesize evidence on biological tapering and treatment discontinuation and its effectiveness in children with AID. In the only randomized, controlled study, Quartier et al., demonstrated that for IL-1 inhibition with canakinumab dose reduction resulted in sustained remission off medication in 45% of sJIA patients compared to only 22% in the interval prolongation arm [17]. Vastert et al., demonstrated that early biological treatment with anakinra was associated with high rates of sustained remission; 73% of sJIA patients were able to successfully discontinue treatment [19].

**Fig. 2:**
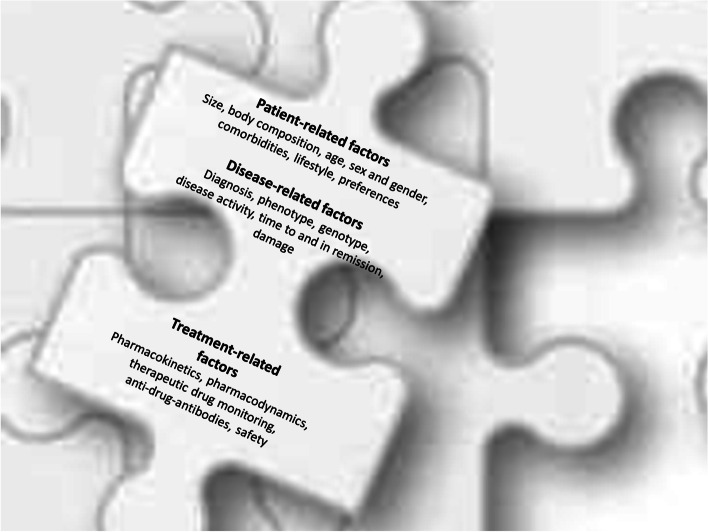
Taper-to-target: Precision health approaches for autoinflammatory diseases

There are data that biological treatment can be tapered after achievement of inactive disease for a certain period. However, data on when to start tapering are inhomogeneous. In all identified studies [16–19], tapering was only started after achievement of inactive disease or sustained CR. Within the group of AID, some diseases (e.g. sJIA) have clear defined inactive disease criteria, whereas others have none, this makes the decision when tapering can be considered challenging. Tapering was only started after a certain period after sustained inactive disease/remission [16–19]. This “safety interval” may be influenced by duration of prior disease activity and time/effort needed to achieve inactive disease. The “safety interval” may be short, particularly in those patients, who had received appropriate treatment during the window of opportunity [21, 22], leading to early inactive disease. Whereas long intervals between disease onset and treatment start may result in higher risk of relapse/flare during tapering [23]. Furthermore, decisions on when to taper are influenced by the safety of the administered drug, and disease characteristics itself [24]. Chhabra et al. reported in their study that sJIA patients have the highest remission frequency off medications (70%), whereas RF + polyarthritis JIA had the lowest (18%) [25]. In addition, special disease characteristics in JIA such as morning stiffness, ankle/wrist involvement, PGA > 30 mm, active joint count > 4, high disease activity, poor patient-reported outcomes before attaining inactive disease are factors which that may compromise the opportunity and success to taper biological treatment [26–28]. However, clinical characteristics associated with increased risks of flare in JIA during tapering were not consistently reported [29]. In AID some additional aspects have to be considered. As AID are mainly life-long chronic conditions, tapering may aim a dose reduction and not treatment discontinuation, particularly in patients with pathogenic gene variants. It is known that pathogenic gene variants will result in high disease activity and risk of disease damage, such as amyloidosis, central nervous complications or hearing loss, whereas variants of unknown significance may have low or no risk of organ damage [30–32]. Therefore, the decision to taper has to consider the genotype. Moreover, the decision when to taper has to address e.g. life circumstances of the AID patient, as stress, infections and cold can trigger flares (Fig. 2).

Tapering in AID should be considered when clinical (e.g. PGA/PPGA, AIDAI) and laboratory (e.g. SAA, S100A12, S100A8/A9) remission is achieved following a personalized “safety interval”. The “safety interval” has to respect disease activity, effort and time to achieve remission, time in remission or inactive disease, as well as phenotype, genotype, comorbidities, organ damage, need for combination treatment, drug safety and personal life events and preferences.

To date, there is evidence to taper biological treatment in children with inflammatory diseases after achievement of inactive disease or sustained CR. All identified studies in this review [16–19] indicate that tapering in sJIA is possible after achievement of sustained inactive disease or CR, irrespective of biological treatment. There is evidence in rheumatic inflammatory diseases, that inactive disease may be sustained with lower drug concentrations than needed to treat active disease [33, 34]. Furthermore, gradual tapering yields inactive disease, and will reduce potential risk for adverse events and costs [35, 36]. However, how to taper most effectively remains uncertain. The identified studies [16–19] tapered biological treatment either by dose reduction or interval prolongation. Only Quartier et al. [17], directly compared both tapering strategies for the same treatment (canakinumab 4 mg/kg/q4w) and the same indication (sJIA). The study demonstrated that 26/38 patients (68%) could be effectively tapered to 1 mg/kg/q4w canakinumab with successful treatment discontinuation in 17/38 patients (45%). In contrast, canakinumab interval prolongation to 4 mg/kg/q12w maintained inactive disease in 30/37 patients (81%). Finally, treatment discontinuation was at least only possible in 8/37 patients (22%). In a Childhood Arthritis Rheumatology Research Alliance (CARRA) survey the physicians’ attitudes towards tapering in sJIA were assessed; 39% preferred tapering biological treatment over 2–6 months and favoured interval prolongation [37]. Particularly for children, interval prolongation often increases quality of life due to a reduced hospitalization rate for infusions or decreased frequency of s.c injections, which are often associated with discomfort, fear and worries [38]. Furthermore, interval prolongation may result in lower drug costs, as absolute treatment doses needed in childhood are commonly lower than those offered in the adult-dose vial, resulting in expensive drug discharge with every injection. The study by Quartier et al. [17] indicates that more high-quality data defining optimal tapering/discontinuation strategies are needed. The question arises, if a paradigm shift from standardized tapering regimens to personalized tapering strategies should be the next step. It is well documented that responses to biological treatment are variable. Specifically, for children with AID personalized treat-to-target strategies are critical for effective disease management [9]. Correspondingly, effective tapering of biological treatment in AID may mandate the development of personalized “taper-to-target” approaches.

These approaches can be enabled by pharmacometric modelling. The mode of action of therapeutic mAb (e.g. TNF-Inhibitors) can be simplified as a dose-concentration-effect relationship. In case of active disease, a high amount of antigen mass has to be expected, leading to a non-linear elimination shape with decreased mAb concentration [39]. In addition, increased body size and presence of anti-drug-antibodies can decrease mAb concentration with decreased treatment response [40]. This indicates that the pharmacokinetic (PK) variability influences -at least in part- the treatment response and may also influence the success of tapering. Consequently, the “taper-to-target” strategies might be guided by pharmacometric modelling, based on PK and pharmacodynamics (PD), disease activity and duration, comorbidities, and safety (Fig. 2). These personalized “taper-to-target” strategies based on PK-PD modelling and/or therapeutic drug monitoring (TDM) might be the more effective and safe, compared to standardized tapering by dose reduction/interval prolongation. However, TDM based therapy control and tapering in AID is currently limited as several assays are not commercially available. Up to now, TDM based successful tapering has been reported for rheumatoid arthritis (RA). In RA tapering without relapse was feasible as long as TNF-inhibitor concentration was high enough to control the antigen mass [41]. Furthermore, TDM guided tapering with model-based algorithms resulted in an overall remission/low disease activity for IV tocilizumab in RA [42]. However, PK data for paediatrics are limited. As children differ from adults in diseases, body composition, age-dependent maturation, and PK [43–45], paediatric PK-PD data are needed. This will result in increased knowledge in PK and TDM, allowing effective personalized “taper-to-target” strategies in the near future.

This study has several limitations. There may be a risk of a reporting bias as unpublished studies were not included and the clinical trials register (www.clinicaltrials.gov) offering additional active research in the field was not reviewed. Furthermore, case reports/studies including a mixed adult-paediatric population without separated data for children and adults were excluded. The very strict inclusion criteria resulted in high quality data, which is summarized in this review. However, data on tapering biological treatment in AID are limited and currently only data in sJIA was identified. Furthermore, it has to be addressed that available tapering studies vary in biological treatment, tapering strategy and safety interval, highlighting the need for standardized prospective trials.

## Conclusion

The available studies indicate evidence of tapering after sustained CR or inactive disease. Up to now, tapering regimens in paediatric AID include dose reduction or interval prolongation, but uncertainty remains on how to taper most effectively. Particularly in AID personalized treat-to-target strategies are essential to achieve inactive disease. Correspondingly, the question arises as to whether a paradigm shift from standardized tapering to personalized “taper-to-target” strategies is necessary, addressing patient related factors (e.g. live circumstances, comorbidities), disease aspects (e.g. severity, genetic variants, damage) and treatment-related factors (e.g. PK, PD, safety). PK-PD/TDM data from high quality prospective tapering studies are urgently needed. Therefore, for the next decade the attention of the rheumatology community should also include clinical trials of safely and effectively tapering and discontinuation of biological treatment. This research will help to develop personalized “taper-to-target” strategies based on PK-PD modelling and/or TDM to assist clinicians in daily patients` care.

## Supplementary Information

**Additional file 1.**

## Data Availability

All data is presented in this manuscript.

## Abbreviations

AIDAI: Autoinflammatory disease activity index
AID: Autoinflammatory Diseases
AOSD: Adult-onset still’s disease
CAPS: Cryopyrin associated periodic syndromes
CARRA: Childhood Arthritis Rheumatology Research Alliance
CR: Clinical remission
CRP: C-reactive protein
FMF: Familial Mediterranean fever
IL: Interleukin
IV: Intravenous
JIA: Juvenile idiopathic arthritis
MKD: Mevalonate kinase deficiency
MTX: Methotrexate
PD: Pharmacodynamics
PGA: Physician’s global assessment
PK: Pharmacokinetic
PPGA: Patients/parent global assessment
RA: rheumatoid arthritis
RF+: Rheumatoid factor positive
SAA: Serum amyloid A
SC: Subcutaneous
sJIA: Systemic juvenile idiopathic arthritis
TDM: Therapeutic drug monitoring
TRAPS: Tumor necrosis factor receptor-associated periodic syndrome
T2T: Treat-to-target
