# Supplementary material for: Tapering of biological treatment in autoinflammatory diseases: a scoping review
Source: Pediatr Rheumatol Online J. 2022 Aug 13;20:67. doi: 10.1186/s12969-022-00725-3 (PMC9375310; doi:10.1186/s12969-022-00725-3)
Supplement: Supplementary file 1 — Additional file 1. [file 12969_2022_725_MOESM1_ESM.docx]

**Additional file 1**

**Additional Table S1: Search Protocol**

| 1. Background | |
| --- | --- |
| A systematic literature review was conducted following the Preferred Reporting Items for Systematic Reviews and Meta-Analyses guidelines [1] to evaluate and summarize the available evidence of biological treatment taper or discontinuation strategies in children and youth with AID and to explore key factors that may inform successful strategies. | |
| 1. Objective | |
| The primary objective is to provide an independent depository of original articles in pediatric and adolescent patients with autoinflammatory disease (AID) and systemic juvenile idiopathic arthritis (sJIA) on biological treatment, describing the taper regimen and the effectiveness of tapering/ treatment discontinuation. | |
| 1. Methods | |
| 3.1 Eligibility | Titles and abstracts are screened for eligibility according to the pre-defined criteria:  A) Eligibility criteria   - - Patients aged 18 years and younger   - Treatment with biological drugs defined as     - IL-1 inhibitors: anakinra, canakinumab, rilonacept     - IL-6 inhibitors: tocilizumab   - Sample size ≥ five patients   - Patients with confirmed AID and sJIA   - Reporting relevant primary or secondary endpoint/outcome of     - Tapering regimen     - Regimen of discontinuation     - Effectiveness of tapering     - Effectiveness of discontinuation   - English language   - Published after 1990   B) Exclusion criteria   - - Indication not relevant   - Population not relevant   - Study design not relevant     - Non-human studies (laboratory or animal studies)     - Non-original articles (reviews, editorials, letters to the editors, expert opinion)   - Treatment not relevant   - Endpoint/outcome not relevant   - Duplicate of prior published results without any additional information |
| 3.2  Search and retrieval | The primary sources of information using the OVID platform for this database were: Embase (1990 to August 2020); MEDLINE Epub ahead of print, In-process and other non-indexed citations (August 2020); MEDLINE without revisions (1996 to August 2020); Evidence-Based Medicine (EBM) Reviews: Cochrane Database of Systematic Reviews (2005 to August 2020); and EBM Reviews: Cochrane Central Register of Controlled Trials (August 2020). |
| 3.3  MESH and search terms | Hereditary autoinflammatory diseases OR cryopyrin associated periodic syndromes (CAPS) OR familial Mediterranean fever (FMF) OR mevalonate kinase deficiency (MKD) OR hyper-IgM immunodeficiency OR systemic juvenile idiopathic arthritis (sJIA) OR arthritis, juvenile OR tumor necrosis factor receptor-associated periodic syndrome (TRAPS) OR adult-onset still’s disease (AOSD) AND treatment OR stopping of treatment AND biological treatment AND therapeutics OR IL-1 inhibitors OR IL-6 inhibitors AND paediatric OR child OR adolescent |
| 3.4 Identification | Identification of eligible study reports were based on MESH and search terms. References list of published studies were reviewed to identify any additional references. The search results were exported and managed in a spreadsheet. |
| 3.5 Screening and eligibility assessment | Initial screening, based on retrieved titles and abstracts, as well as an eligibility assessment of selected full-text publications were performed by two scientists. Reasons for exclusion were listed. |
| 3.6  Quality assessment | All included full-text papers were checked for quality. A score of 1 was given for each applicable question, with a maximum total score of 6. Articles with a total score of 4 or higher and at least 2 of 4 essential questions were rated high quality. In addition, selected articles were evaluated with the Oxford Levels of Evidence [2]. For details please refer to additional Table S2. |
| 3.7 Data extraction | Data extraction was done by scientists using a manual data extraction spreadsheet developed in MS Excel. Consensus sessions were held regularly to resolve data extraction issues. |
| 3.8 Final inclusion | Final selection of the study reports was performed after the scientists involved in the project reached consensus. |

**Additional Table S2: Quality assessment of selected studies**

| **Item assessed** | **Coding framework** | | |
| --- | --- | --- | --- |
| **Appropriate methods to select participants** | | | |
| 1. Sampling frame, age and sex of sample described | Yes | No | Don’t know |
| 1. >80% participation or comparison of consents and refusals* | Yes | No | Don’t know |
| **Appropriate methods to measure treatment strategies** | | | |
| 1. Treatment strategy reproducible* | Yes | No | Don’t know |
| **Appropriate methods to measure tapering/stopping of**  **treatment strategies** | | | |
| 1. Reasons for tapering/stopping reproducible* | Yes | No | Don’t know |
| 1. Method of tapering/stopping reproducible* | Yes | No | Don’t know |
| **Conflict of interest** | | | |
| 1. Conflict of interest declaration | Yes | No | Don’t know |

Assessment modified from Sanderson [3] and Pasma [4], * designated essential question

**References**

1. Moher, D., et al., *Preferred reporting items for systematic reviews and meta-analyses: the PRISMA statement.* Ann Intern Med, 2009. **151**(4): p. 264-9, W64.

2. Phillips, B., *Oxford Center for Evidence-based Medicine. Levels of Evidence, March 2009.* <http://www>. cebm. net/index. aspx? o= 1025, 2009.

3. Sanderson, S., I.D. Tatt, and J.P. Higgins, *Tools for assessing quality and susceptibility to bias in observational studies in epidemiology: a systematic review and annotated bibliography.* International Journal of Epidemiology, 2007. **36**(3): p. 666-676.

4. Pasma, A., et al., *Factors associated with adherence to pharmaceutical treatment for rheumatoid arthritis patients: a systematic review.* Semin Arthritis Rheum, 2013. **43**(1): p. 18-28.
